# Effects of quercetin and coated sodium butyrate dietary supplementation in diquat-challenged pullets

**DOI:** 10.5713/ab.21.0493

**Published:** 2022-03-03

**Authors:** Ning Zhou, Yong Tian, Wenchao Liu, Bingjiang Tu, Tiantian Gu, Wenwu Xu, Kang Zou, Lizhi Lu

**Affiliations:** 1 College of Animal Science and Technology, Nanjing Agricultural University, Nanjing 210095, China; 2 State Key Laboratory for Managing Biotic and Chemical Threats to the Quality and Safety of Agro-products, Institute of Animal Science & Veterinary, Zhejiang Academy of Agricultural Sciences, Hangzhou, 310000, China; 3 Huzhou Lvchang Ecoagriculture Co., Ltd. Huzhou, 313025, China; 4 Huzhou Wuxing District Animal Disease Prevention and Control Center, Huzhou, 313000, China

**Keywords:** Pullets, Quercetin, Coated Sodium Butyrate, Oxidative Stress, Small Intestine

## Abstract

**Objective:**

This study was designed to investigate the hypothesis that dietary quercetin (QUE) and coated sodium butyrate (SB) supplementation alleviate oxidative stress in the small intestine of diquat (DIQ)-challenged pullets.

**Methods:**

A total of 200 13-week-old pullets were divided into four groups: the control group (CON), the DIQ group, the QUE group, and the coated SB group, and injected intraperitoneally with either saline (CON) or diquat (DIQ, QUE, and SB) to induce oxidative stress on day 0.

**Results:**

On the first day, the malondialdehyde and superoxide dismutase (SOD) concentrations in the SB group were significantly different from those in the DIQ and QUE groups (p<0.05), and dietary supplementation with SB increased serum glutathione peroxidase (GSH-PX) levels compared with the DIQ group (p<0.05). Quercetin and SB increased the levels of *CLAUDIN-1* and zonula occludens-1 (*ZO-1*) in the jejunum. On the tenth day of treatment, QUE attenuated the decrease in GSH-PX levels compared to those of the CON group (p<0.05), while SB increased SOD, GSH-PX, and total antioxidant capacity levels compared to those of the DIQ group. Nuclear factor erythroid 2-related factor 2 (*NRF2*) and heme oxygenase-1 (*HO-1*) mRNA levels in the QUE and SB groups increased (p<0.05) and *CLAUDIN-1* mRNA levels in the QUE and SB groups were upregulated compared to those in the DIQ group ileum tissue.

**Conclusion:**

Supplementation of QUE and SB demonstrated the ability to relieve oxidative stress in pullets post DIQ-injection with a time-dependent manner and QUE and SB may be potential antioxidant additives for relieving oxidative stress and protecting the intestinal barrier of pullets.

## INTRODUCTION

Intensive modern farming aims to boost livestock productivity and economic benefits; however, the focus on increased production over animal welfare also increases the risk of animal exposure to oxidative stress [1]. Oxidative stress, which represents the imbalance between oxidant and antioxidant intracellular systems, is considered to play a significant role in regulating the metabolic activity of some organs and productivity in farm animals, so increased stress leads to reduced production [2]. Imbalance of factors like physics/chemistry, nutrition, temperature, and environment can induce oxidative stress with increasing production of the reactive species malondialdehyde (MDA), causing a change in antioxidant defense capacity, including superoxide dismutase (SOD), total antioxidant capacity (T-AOC), catalase, and glutathione peroxidase (GSH-PX) [3]. Nuclear factor erythroid 2-related factor 2 (NRF2), which belongs to the Keap-ARE system, is a transcription factor with an important regulatory effect on oxidative status through induction of the expression of antioxidant and phase 2 detoxifying enzymes [4]. In addition, NAD (P) H dehydrogenase quinone 1 (NQO1) and heme oxygenase-1 (HO-1) play key roles in the Keap-ARE system. Results have shown that oxidative stress and disruption of cellular redox status impair intestinal function [5]. The formation of tight junctions is one of the major components of the intestinal barrier. Occludin was the first identified tight junction protein, whereas claudins and zonula occludens-1 (ZO-1) are the main proteins that contribute to the physiological and structural paracellular barrier function [6]. Meanwhile, it is imperative to establish appropriate nutritional strategies to decrease the risk of intestinal oxidative damage in pullets.

Diquat (DIQ) is widely used to induce oxidative stress in animals [7–9]. Diquat-induced oxidative stress has a negative effect on jejunal and ileal morphology and can increase the histopathological grading of the jejunum, ileum, and colon in piglets [10]. Quercetin (QUE), a flavonoid compound, is widely distributed in plants and has been reported to possess strong anti-inflammatory, antioxidant, and antibacterial effects [11,12]. Quercetin has been shown to alleviate oxidative stress by scavenging free radicals, removing oxidation products, and stimulating antioxidant enzymes in many animals [13,14]. Butyrate and its sodium salts are known to modulate immune and inflammatory responses and promote intestinal integrity [15,16]. Previous studies have shown that sodium butyrate (SB) has beneficial effects on growth performance, gut morphology, and anti-microbial, immunomodulatory, and anti-oxidative capacities [17,18]. In broiler chickens, supplementation with SB increases growth, performance, intestinal histomorphology, antioxidant status, expression of gut leakiness indicators, and inflammatory cytokines [19]; however, there are few known results in pullets.

Therefore, the objective of this study was to evaluate the influence of QUE and SB on oxidative stress-induced impairments, serum anti-oxidative capacity, intestinal morphology, and mRNA expression levels of the NRF2 pathway and tight junction-related genes in pullets. We hypothesized that QUE and SB could alleviate DIQ-induced oxidative stress and intestinal barrier dysfunction in pullets.

## MATERIALS AND METHODS

### Animals, diets, and management

This trial was carried out in Huzhou Lvchang Ecoagriculture Co., Ltd., Zhejiang Province, in August. A total of 200 healthy Jingfen No.1 chickens (1.15 kg±0.16) at 13 weeks of age were recruited and randomly assigned into four equal dietary treatments with five replicates, ten pullets per replicate: a control group fed a basal diet (CON), a diquat-treated group fed a basal diet (DIQ), QUE group fed a basal diet containing 500 mg/kg QUE, and SB group fed a basal diet containing 500 mg/kg coated sodium butyrate. Diquat was purchased from Shangdong Baishiwei Crop Protection Co., Ltd. (Weifang, Shandong, China). Quercetin was purchased from Chengdu Ookang Pharmaceutical Co., Ltd., and coated sodium butyrate was purchased from Hangzhou KingTechina Feed Co., Ltd (Hangzhou, Zhejiang, China). All birds were fed the respective diets for 7 days and then injected intraperitoneally with either 1 mL saline (CON) or 8 mg/kg body weight diquat (DIQ, QUE, and SB) in 1 mL saline to induce oxidative stress. The dose of DIQ was referenced to previous study [8]. Pullets were caged in 3-tier battery cages, with five chickens per pen with dimensions of 40 cm×50 cm×40 cm. Diet and fresh water were offered *ad libitum*, and the light regimen was set to 16 L:8 D during the experimental period. The temperature, humidity, and light conditions of all groups were the same. The basal diets provided to meet the nutritional requirements of hens are listed in Table 1. All procedures were implemented according to the Local Experimental Animal Care Committee and approved by the ethics committee of Nanjing Agricultural University (NO.SYXK(SU) 2011-0036).

### Sample collection

The trial lasted seventeen days, with the day of intraperitoneal injected considered day 0. On the first and tenth days, one pullet was selected from each group replicate; therefore, a total of 40 pullets were sampled. Then, the chickens were slaughtered after fasting for 12 hours, and blood, jejunum, and ileum samples were collected. Blood samples, collected into vacuum tubes (5 mL) containing coagulant, were centrifuged at 3,000×g for 15 min at 4°C, and the serum was stored at −20°C for further analysis. Part of the jejunum and ileum samples were immediately frozen in liquid nitrogen and stored at −80°C for further analysis. Another part of the intestinal samples (tenth day) was fixed in 4% paraform (Biosharp, Shanghai, China) and stored at room temperature for histological examination.

### Estimation of serum antioxidant parameters

The T-AOC, SOD, GSH-PX activity, and MDA concentrations in serum samples were measured to estimate the oxidative status. Antioxidant parameter levels were determined using T-AOC (HY-60021), SOD (HY-M0001), GSH-PX (HY-60005), and MDA (HY-60003) assay kits from the Beijing Sino-UK Institute of Biological Technology, according to manufacturer instructions.

### Histological assay

Jejunum and ileum samples were prepared for histological analysis, and the samples treated with formaldehyde solution were embedded in paraffin, cut into 5-μm-thick sections, and subjected to hematoxylin-eosin (H&E) staining using the standard procedures of dehydration, embedding, sectioning, and staining [10]. Villous height and crypt depth were measured from three discontinuous sections of each sample were made for observation, and six complete, typical fields of view were selected for each sample using an optical binocular microscope (Olympus BX5; Olympus Optical Co. Ltd, Tokyo, Japan) equipped with a digital camera (Nikon Eclipse Ci-L; Nikon, Tokyo, Japan) and an image analyzer (Image Pro Plus 6.0; Media Cybernetics, Bethesda, MD, USA).

### Total RNA isolation and quantitative real-time polymerase chain reaction

Total RNA was extracted from different tissues using the E.Z.N.A Total RNA Kit II (OMEGA Bio-tek, Norcross, GA, USA) according to manufacturer instructions, and then stored at −80°C for cDNA synthesis. RNA quality was measured using a Nanodrop system (Thermo Fisher, Waltham, MA, USA) at 260 nm and 280 nm. Polymerase chain reaction (PCR) amplifications of *NRF2*, *NQO1*, *HO-1*, CLAUDIN-1, OCCLUDIN, and *ZO-1* cDNA were expressed in all jejunal and ileal samples. All primer sequences (Table 2) were designed using the Primer 5.0. The PCR experiments were performed with the LightCycler96 (Roche, Basel, Switzerland) and SYBR Green PCR Master Mix (Vazyme, Q711-02/03, Nanjing, China) according to the manufacturers’ protocols. The reaction was amplified at 95°C for 3 min, followed by 40 cycles of 95°C for 30 s and 60°C for 30 s. For each sample, three replicates were performed, and all reactions were run in triplicate.

### Statistical analysis

The data was collected from each reaction, and the relative expression levels of different gene mRNAs to *β-actin* mRNA were evaluated using the 2^–ΔΔCT^ method [20]. Data were analyzed by one-way analysis of variance followed by least significant difference post hoc test and were reported as the mean±standard error of means. Statistical analyses were performed using SPSS 15.0. Statistical significance was set at p<0.05.

## RESULTS

### Serum antioxidative capacity

As shown in Table 3, on the first post-injection day, serum MDA content in the DIQ group was higher than that in both the CON and SB groups (p<0.05). However, the MDA concentration in the QUE group was not different from that in the DIQ group. Diquat treatment tended to block serum SOD activity (p<0.05) and sodium butyrate treatment markedly restored (p<0.05) inhibitory function, compared to the DIQ and QUE treatments. GSH-PX concentration significantly decreased in the DIQ group (p<0.05), and dietary supplementation with sodium butyrate increased serum GSH-PX levels compared to the DIQ group (p<0.05). The serum T-AOC level was greater in SB chickens on DIQ treatment than in the DIQ group (p<0.05). Moreover, the antioxidant status of serum was examined on tenth day after DIQ injection (Table 3). Compared with CON group, the DIQ significantly increased the serum MDA concentration and decreased both SOD and GSH-PX as well as the T-AOC concentration; QUE attenuated the decrease in GSH-PX levels induced by DIQ (p<0.05), and sodium butyrate upregulated T-AOC levels (p<0.05) and increased SOD and GSH-PX levels compared with the DIQ group.

### Intestinal morphology

**Figure 1:**
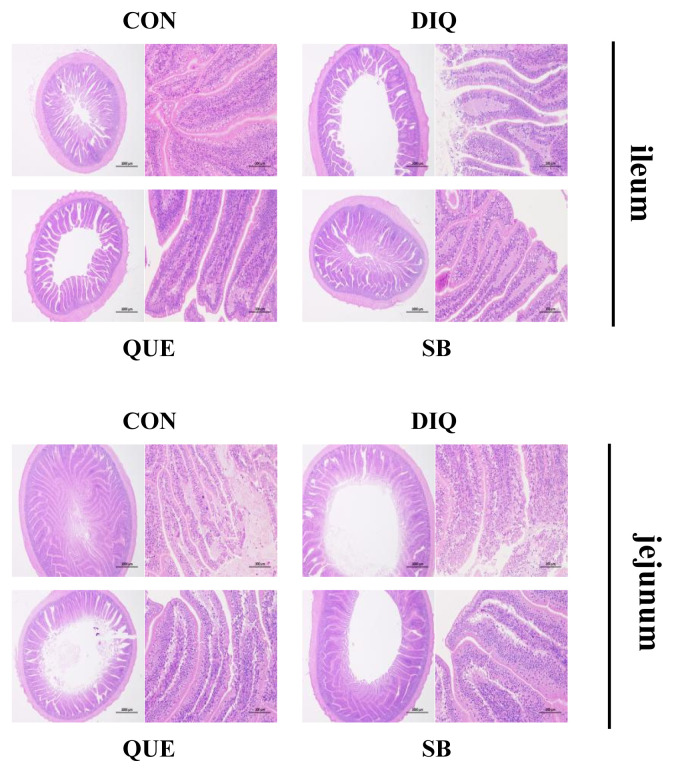
Effect of quercetin and sodium butyrate on intestinal morphological structure in diquat-induce oxidative stress with pullets on tenth day (HE, ×20 and ×200). CON, pullets fed basal diet; DIQ, diquat-injection pullets; QUE, pullets fed with a basal diet containing quercetin and injected with diquat; SB, pullets fed with a basal diet containing sodium butyrate and injected with diquat.

Morphology, villus height, and crypt depth of the jejunum and ileum on the tenth day is showed in Figure 1 and Table 4. In the ileum tissue, the structure of the CON group tissue was clear, the mucosal layer epithelium was complete, the number of intestinal glands was abundant, and the intestinal villi were arranged regularly. In the DIQ group, the villus height was higher (p<0.05) and many epithelial cells were necrotic compared to the CON group. In the SB group, the villus height was higher (p<0.05) and the intestinal villi were arranged regularly with little shedding compared to the DIQ group. Similar to the ileum morphology phenomenon, in jejunum tissue, DIQ treatment resulted in a large number of exfoliated or absent intestinal villous epithelial cells compared to the DIQ group (p<0.05), while villus height of the SB group was higher than that of the DIQ group (p<0.05). Quercetin treatment did not appear to reduce the ileum or jejunum injury post-diquat treatment.

### Relative mRNA expression of NRF2 signaling pathway and tight junction mRNAs in ileum tissue

**Figure 2:**
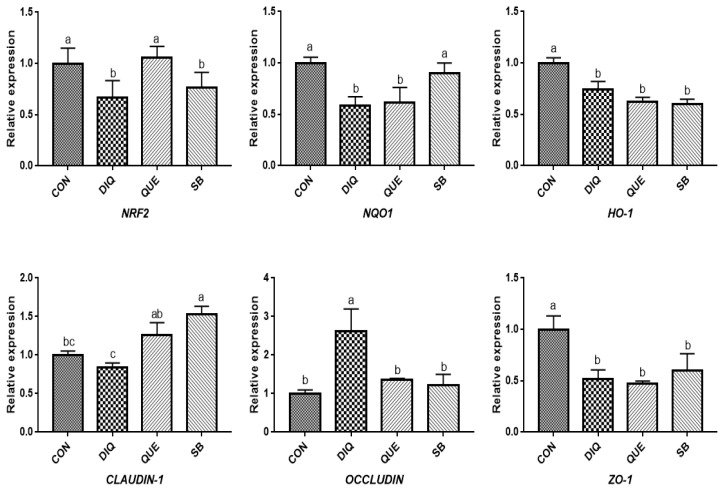
Effects of dietary quercetin and sodium butyrate supplementation on mRNA expression of antioxidant genes and tight junction RNAs in ileum on first day. *NRF2*, nuclear factor erythroid 2-related factor 2; *NQO1*, NAD (P) H dehydrogenase quinone 1; *HO-1*, heme oxygenase-1; *ZO1*, zonula occludens-1. CON, pullets fed basal diet; DIQ, diquat-injection pullets; QUE, pullets fed with a basal diet containing quercetin and injected with diquat; SB, pullets fed with a basal diet containing sodium butyrate and injected with diquat. ^a–c^ Data are means±standard error; mean values sharing different lowercase letters differ significantly (p<0.05).

**Figure 3:**
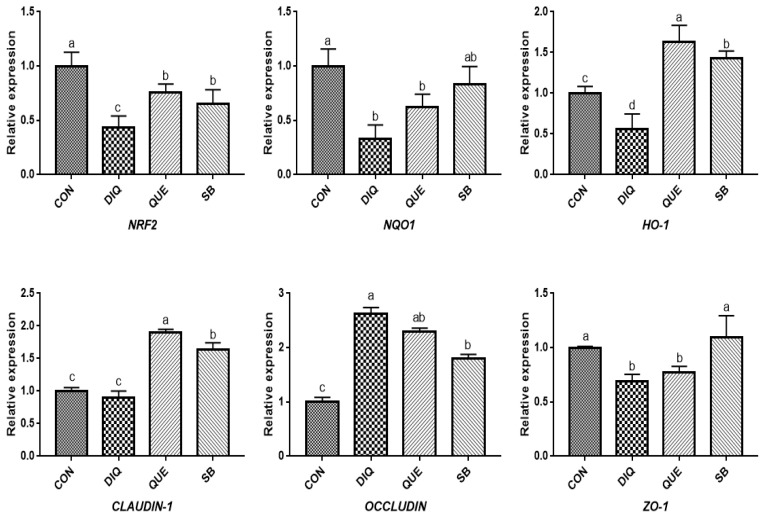
Effects of dietary quercetin and sodium butyrate supplementation on mRNA expression of antioxidant genes and tight junction RNAs in ileum on tenth day. *NRF2*, nuclear factor erythroid 2-related factor 2; *NQO1*, NAD (P) H dehydrogenase quinone 1; *HO-1*, heme oxygenase-1; *ZO1*, zonula occludens-1. CON, pullets fed basal diet; DIQ, diquat-injection pullets; QUE, pullets fed with a basal diet containing quercetin and injected with diquat; SB, pullets fed with a basal diet containing sodium butyrate and injected with diquat. ^a–d^ Data are means±standard error; mean values sharing different lowercase letters differ significantly (p<0.05).

According to the day one measurements, the relative mRNA expression of the NRF2 signaling pathway is shown in Figure 2. Compared with the CON group, DIQ-treated birds exhibited lower mRNA levels of *NRF2*, *NQO1*, and *HO-1* in the ileum (p<0.05), whereas DIQ-challenged birds in the QUE group exhibited significantly increased levels of *NRF2* mRNA expression compared to those of the DIQ group (p<0.05). *NQO1* mRNA expression in the SB group was higher than that in the DIQ group (p<0.05). The mRNA expression levels of *CLAUDIN-1*, *OCCLUDIN*, and *ZO-1* in the ileum are presented in Figure 2. *CLAUDIN-1* mRNA expression in the CON group was similar to that of the DIQ group; however, dietary supplementation with QUE and sodium butyrate was associated with upregulated expression of *CLAUDIN-1* in the ileum (p<0.05). The mRNA expression levels of *OCCLUDIN* were significantly increased in the DIQ group compared with the CON group, but the supplementation groups did not exhibit this phenomenon. Next, we performed RT-PCR to test the expression of *NRF2*, *NQO1*, *HO-1*, *CLAUDIN-1*, *OCCLUDIN*, and *ZO-1* mRNA in the ileum of the four groups on the tenth day (Figure 3). Compared with the CON group, the expression of *NRF2*, *NQO1*, and *HO-1* was significantly lower in the DIQ group, while *NRF2* and *HO-1* mRNA levels in the QUE and SB groups were higher (p<0.05). In addition, the *CLAUDIN-1* mRNA levels in the QUE and SB groups were higher than those in the DIQ group (p<0.05).

### Relative mRNA expression of NRF2 signaling pathway and tight junction mRNAs in jejunum tissue

**Figure 4:**
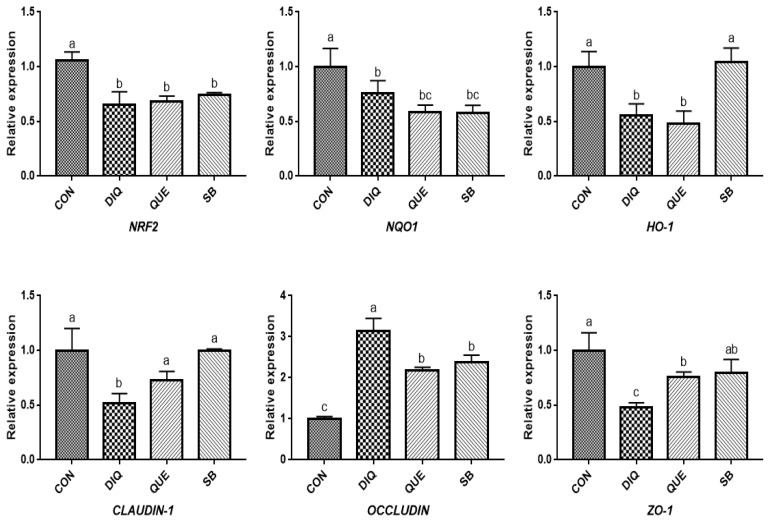
Effects of dietary quercetin and sodium butyrate supplementation on mRNA expression of antioxidant genes and tight junction RNAs in jejunum on first day. *NRF2*, nuclear factor erythroid 2-related factor 2; *NQO1*, NAD (P) H dehydrogenase quinone 1; *HO-1*, heme oxygenase-1; *ZO1*, zonula occludens-1. CON, pullets fed basal diet; DIQ, diquat-injection pullets; QUE, pullets fed with a basal diet containing quercetin and injected with diquat; SB, pullets fed with a basal diet containing sodium butyrate and injected with diquat. ^a–c^ Data are means±standard error; mean values sharing different lowercase letters differ significantly (p<0.05).

**Figure 5:**
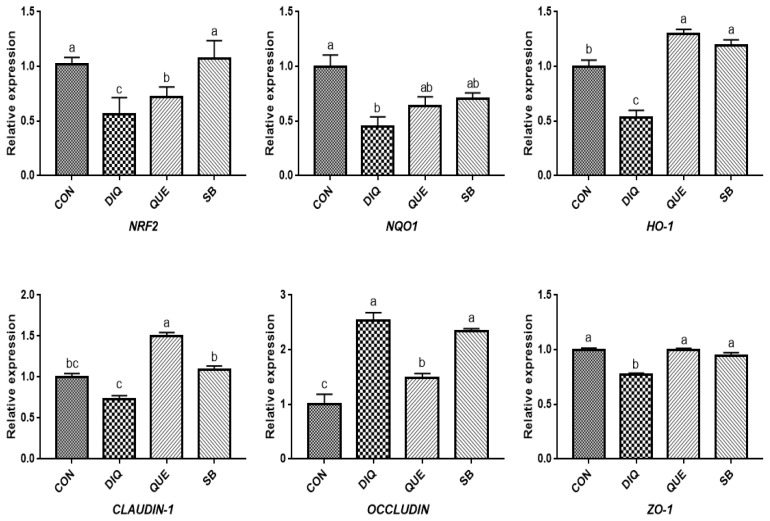
Effects of dietary quercetin and sodium butyrate supplementation on mRNA expression of antioxidant genes and tight junction RNAs in jejunum on tenth day. *NRF2*, nuclear factor erythroid 2-related factor 2; *NQO1*, NAD (P) H dehydrogenase quinone 1; *HO-1*, heme oxygenase-1; *ZO1*, zonula occludens-1. CON, pullets fed basal diet; DIQ, diquat-injection pullets; QUE, pullets fed with a basal diet containing quercetin and injected with diquat; SB, pullets fed with a basal diet containing sodium butyrate and injected with diquat. ^a–c^ Data are means±standard error; mean values sharing different lowercase letters differ significantly (p<0.05).

According to measurements on the first day (Figure 4), the results showed a downregulation trend of *NRF2*, *NQO1*, and *HO-1* mRNA levels when exposed to DIQ (p<0.05). The *NRF2* and *NQO1* mRNA levels in the DIQ group were not significantly different from those in the QUE and SB groups (p> 0.05), while the *HO-1* mRNA level in the SB group was significantly higher than that in the DIQ group. Compared with the CON group, DIQ group showed lower (p<0.05) mRNA abundance of *CLAUDIN-1* and *ZO-1* mRNA, while SB and QUE groups showed higher expression levels (p<0.05). The relative mRNA expression of the NRF2 signaling pathway and tight junction (*NRF2*, *NQO1*, *HO-1*, *CLAUDIN-1*, *OCCLUDIN*, and *ZO-1*) on day are shown in Figure 5. Unlike on day one, *NRF2* and *HO-1* mRNA levels of the QUE and SB groups were higher than those of the DIQ group on day 10. The *NQO1* expression in the QUE and SB groups exhibited increasing trends over time compared with DIQ group. *CLAUDIN-1* and *ZO-1* mRNA expression in the DIQ group was downregulated overall compared to that in the CON group. In contrast, the QUE and SB groups had higher expression levels of *OCCLUDIN* and *ZO-1* than the DIQ group.

## DISCUSSION

Diquat-induced oxidative stress has been reported to affect intestinal morphology and antioxidant capacity in animals and play a principal role in disrupting intestinal function and breaking oxidative balance [9,10,20]. In this investigation, we observed that serum MDA concentration of samples was increased in DIQ group on the first and tenth day. The other main anti-oxidative parameters, SOD, GSH-PX, and T-AOC, in the CON group were lower in the DIQ group on the first and tenth days, which was consistent with previous reports [8–10]. Quercetin is one of the most widely distributed bioflavonoids and exhibits metal chelation and free-radical scavenging activities to fight oxidative stress [21]. A previous study demonstrated that supplementation with QUE could relieve the upregulation of MDA and downregulation of GSH-PX and SOD in jejunal tissue [14]. Quercetin treatment was shown to be beneficial for decreasing MDA levels in transported pigs [22]. These previous results are similar to the observations made on the tenth day of the present study. Butyric acid has been shown to play an important role in maintaining the integrity of the intestinal mucosa and exerting potent antioxidative effects in animals [19]. A study found that serum GSH-PX activity of calves linearly increased and serum MDA concentration linearly decreased after sodium butyrate supplementation during the entire experimental period [23], which is similar to our results. In addition, serum MDA levels were significantly lower after sodium butyrate supplementation on the first day, which was noted previously in stressed broiler chickens supplemented with microencapsulated NaB [18].

The intestinal epithelial barrier is the first line of defense against hostile environments within the intestinal lumen. This study found that DIQ injection disrupted the intestinal morphology, resulting in large-scale shedding of intestinal villi, and high incidence of necrotic epithelial cells, which was similar to the findings of a few previous studies [7,20,24]. Our results were similar to those of a previously examined diet containing 500 mg/kg QUE, which had no significant effect on villus height, crypt depth, or ratio of villus height to crypt depth in the jejunum and ileum under stress induced by lipopolysaccharide [14].

NRF2, a pivotal sensor of oxidative stress, is a transcription factor that plays a central role in the regulation of antioxidant and phase 2 detoxifying enzymes and related proteins, and has been reported to regulate the antioxidant response and represent the underlying mechanism that provides a pivotal defense against DIQ toxicity [25]. Once stimulated by inducers, NRF2 activates downstream enzymes, including NQO1 and HO-1, to prevent oxidative stress damage. In the present study, DIQ treatment caused downregulated intestinal *NRF2* expression and its downstream target genes *NQO1 HO-1*, which can be considered an acute response to DIQ-induced oxidative stress, which is in agreement with previous results [24,26]. Previous studies have shown that supplementation with QUE attenuates the lipopolysaccharide-induced reduction of *HO-1* and *NQO1* mRNA levels in jejunum tissue [14] and activates the NRF2 signaling pathway to induce HO-1 expression and protect against oxidative stress [12]. Additionally, QUE has been found to effectively inhibit manganese-induced oxidative stress in neural-tumor epithelial cells (SK-N-MC) in Sprague-Dawley rats by upregulating HO-1 and Nrf2 proteins [27]. Our findings showed that QUE promoted *NRF2* and *HO-1* expression levels in the jejunum and ileum on the tenth day compared with the DIQ group, which is consistent with previous findings, suggesting that QUE inhibited DIQ-induced intestinal oxidative stress through the NRF2 signaling pathway. In the present study, we observed upregulation of *NRF2* in the ileum and *NQO1* on the tenth day, which is similar to a previous study in which the mRNA expression of *NQO1* and *NRF2*, as well as total NRF2 protein levels, were increased in goats fed a sodium butyrate diet [28]. The intestinal barrier is physically composed of epithelial cells connected by tight junction proteins, such as ZO-1, CLAUDIN-1, and OCCLUDIN, and regulation of the selective permeability between epithelial cells and gene expression is important for maintaining the proper functioning of the intestinal epithelial barrier [29]. Tight junction mRNAs and protein expressions varied in response to DIQ exposure. Diquat-induced oxidative stress causes a decrease in tight junction genes expression, leading to increased intestinal permeability, weakening intestinal barrier function and disrupting intestinal structural integrity the structural integrity of the intestine [7,8]. Diquat treatment decreased protein levels of OCCLUDIN, CLAUDIN-1, and ZO-1 in the jejunal mucosa [7]. However, this study found that the mRNA expression of *OCCLUDIN* was significantly increased and that *CLAUDIN-1* and *ZO-1* levels were significantly decreased in the jejunum and ileum of the DIQ group. Whether this phenomenon was induced by DIQ still requires further investigation and may be related to a compensatory effect. Quercetin improved gut barrier function, the QUE-supplemented showed increased *OCCLUDIN* and *ZO-1* mRNA expression in the jejunum of pigs after transport stress [22], different stress patterns may account for differential expression of *CLAUDIN-1* and *OCCLUDIN*. One report have investigated the effect of sodium butyrate on tight junction proteins, and the mRNA and protein expression of CLAUDIN-1 and ZO-1 in goat ruminal epithelium increased after sodium butyrate supplementation, suggested that sodium butyrate could reverse the damage of rumen epithelium tight junction by inhibiting MAPK signaling pathways [30]. Differences of related genes expression between jejunum and ileum may be due to additives working in different parts of the small intestine [26]. Collectively, the present findings provide important evidence of the potential protective effects of QUE and coated sodium butyrate against DIQ-induced dysfunction in the intestinal morphology and barrier of pullets.

## CONCLUSION

In conclusion, supplementation of QUE and sodium butyrate demonstrated the ability to relieve oxidative stress by changing in serum oxidative stress parameters (MDA, SOD, GSH-PX, and T-AOC), interfering with the expression of tight junction mRNAs (*CLAUDIN*, *OCCLUDIN*, *ZO-1*), and modulating NRF2 pathway-related mRNAs (*NRF2*, *HQO1*, *HO-1*) under DIQ stress in pullets in a time-dependent manner, indicating that QUE and sodium butyrate may be potential antioxidant additives for relieving oxidative stress and protecting the intestinal barrier of pullets.

## Figures and Tables

**Table 1:** Ingredients and nutrient composition of the basal diet.

Ingredients	Content (%)	Nutrient levels	Content
Corn	61.03	Metabolic energy^2)^ (MJ/kg)	11.20
Soybean meal	32.52	Crude protein (%)	16.35
Wheat bran	2.00	Lysine (%)	0.87
Soybean oil	0.45	Cysteine+methionine (%)	0.68
Vitamin-mineral premix^1)^	4.00	Calcium (%)	3.50
Total	100	Available phosphorus (%)	0.37

1) The premix provided the following nutrients per kilogram of diet: Vit A, 200,000 IU; Vit D_3_, 80,000 IU; Vit E, 600 IU; Vit K_3_, 62 mg; Vit B_1_, 50 mg; Vit B_3_, 150 mg; Vit B_6_, 90 mg; Vit B_12_, 0.5 mg; niacin, 800 mg; pantothenic acid, 350 mg; folic acid, 30 mg; biotin, 6 mg; choline chloride, 7,800 mg; Fe, 1,500 mg; Cu, 250 mg; Mn, 65 mg; Zn, 1900 mg; Se, 5.8 mg; I, 23 mg.

2) Values are calculated.

**Table 2:** Characteristics of the primers used for the real-time polymerase chain reaction analysis

Genes	Primer (from 5′ to 3′)	Products size (bp)	Accession number
*NRF2*	F: GAGCCCATGGCCTTTCCTAT R: CACAGAGGCCCTGACTCAAA	212	NM_001007858.1
*NQO1*	F: TCGCCGAGCAGAAGAAGATTGAAG R: CGGTGGTGAGTGACAGCATGG	192	NM_001277620.1
*HO-1*	F: AAGAGCCAGGAGAACGGTCA R: AAGAGCCAGGAGAACGGTCA	121	NM_205344
*Claudin-1*	F: GCATGGAGGATGACCAGGTGA R: GAGCCACTCTGTTGCCATACCAT	117	NM_001013611.2
*Occludin*	F: GCAGATGTCCAGCGGTTACTAC R: CGAAGAAGCAGATGAGGCAGAG	176	NM_205128.1
*ZO-1*	F: AAGTGTTTCGGGTTGTGGAC R: GCTGTCTTTGGAAGCGTGTA	160	XM_413773.4
*β-actin*	F: CACCACAGCCGAGAGAGAAAT R: TGACCATCAGGGAGTTCATAGC	135	L08165

F, forward primer; R, reverse primer; *NRF2*, nuclear factor erythroid 2-related factor 2; *NQO1*, NAD (P) H dehydrogenase quinone 1; *HO-1*, heme oxygenase-1; *ZO1*, zonula occludens-1.

**Table 3:** Serum antioxidative status activity of pullets fed with quercetin and sodium butyrate with injected diquat on first day and tenth day

Item	CON^1)^	DIQ^1)^	QUE^1)^	SB^1)^	SEM	p-value
Day 1						
MDA	3.69^b^	4.57^a^	4.90^a^	3.62^b^	0.28	<0.001
SOD	71.72^a^	53.03^b^	53.87^b^	65.64^ab^	3.96	0.009
GSH-PX	666.01^b^	573.90^c^	564.89^c^	835.54^a^	54.38	<0.001
T-AOC	10.72^a^	8.74^b^	8.14^b^	9.97^a^	0.51	<0.001
Day 10						
MDA	2.88^b^	3.47^a^	2.29^c^	3.21^ab^	0.22	0.003
SOD	88.29^a^	71.24^b^	71.08^b^	75.96^b^	3.50	0.002
GSH-PX	1,034.91^a^	930.17^b^	1,037.41^a^	1,002.44^ab^	21.64	0.002
T-AOC	13.52^a^	11.42^b^	12.07^ab^	13.14^a^	0.42	<0.001

SEM, standard error of means; MDA, malondialdehyde (nmol/mL); SOD, superoxide dismutase (U/mL); GSH-PX, glutathione peroxidase (U/mL); T-AOC, total antioxidant capacity (U/mL).

1) CON, pullets fed basal diet; DIQ, diquat-injection pullets; QUE, pullets fed with a basal diet containing quercetin and injected with diquat; SB, pullets fed with a basal diet containing sodium butyrate and injected with diquat.

a–c Means with a different superscript within a row differ significantly (p<0.05).

**Table 4:** Effects of quercetin and sodium butyrate on intestinal morphology in ileum and jejunum of pullets with injected diquat on tenth day

Items	CON^1)^	DIQ^1)^	QUE^1)^	SB^1)^	SEM	p-value
Ileum						
Villus height (μm)	1,166.82^a^	743.52^c^	728.67^c^	1,053.01^b^	95.63	<0.001
Crypt depth (μm)	201.33^a^	141.44^b^	161.42^b^	159.97^b^	10.92	0.025
Villus heigh/crypt depth	5.90^ab^	5.49^ab^	4.72^b^	6.64^a^	0.35	0.031
Jejunum						
Villus height (μm)	1,437.62^a^	971.84^c^	704.82^d^	1,138.78^b^	132.92	<0.001
Crypt depth (μm)	197.47^a^	138.90^b^	111.25^c^	183.79^a^	17.27	<0.001
Villus heigh/crypt depth	7.27^a^	7.07^ab^	6.35^b^	6.35^b^	0.21	0.029

SEM, standard error of means.

1) CON, pullets fed basal diet; DIQ, diquat-injection pullets; QUE, pullets fed with a basal diet containing quercetin and injected with diquat; SB, pullets fed with a basal diet containing sodium butyrate and injected with diquat.

a–d Means with a different superscript within a row differ significantly (p<0.05).
